# Where Do Our Patients Come In?

**Published:** 1913-01-15

**Authors:** H. F Dickason


### WHERE DO OUR PATIENTS COME IN?

BY H. F DICKASON, D.D.S.

It is with pleasure that I read each month the *Digest*, especially Brother Bill's Letters, and other discussions on the financial side of dentistry. There are, however, a few things of a similar nature in all these discussions which I have never been able to thoroughly understand, as well as some things we read on dental subjects which have no relation to the financial side of practice. One of the principal thoughts suggested is, Where do our patients come in? Is it that we are the complete dictators and can do, say, and charge as we please, while our patients passively submit? For instance, one operator will describe an operation in which he has whittled upon the human anatomy as if he were a sculptor working on a piece of marble, but he neglects to describe the acrobatic contortions of said anatomy during the operation. In the August *Digest* there is a caption as follows: "What would you do with this Child?" When I read that article, I could not help likening this dentist's experience with so many of my own, and wonder how these sculptors would handle a case of this kind. It is a fact that few mention, that the patient is a factor to be reckoned with when we are cleaning enamel, making flat bases and steps for fillings, and this reckoning is sometimes so unsatisfactory that we are reminded of the caption, "What would you do with this Child?" I am quite sure that many of us, who are not familiar with "Brownstone Dentistry"—which seems to mean, do as you please, just when you please, and charge what you please—would like to know what kind of hypnotic suggestion is used to enable one to do this. Where do our nervous patients come in; or, is it a fact, that the majority of those who write for the journals practise in that class of society who "have no nerves"? It would be a novel departure and interesting reading to a large body of everyday dentists who practise upon everyday normal people, "who can stand anything except having their teeth worked on," to hear something of what the patient did, and what became of the flat base, bevel walls, steps, etc.

Now in regard to fees: It is easy to figure just how much an hour it costs us to run our office, how much we wish for household expenses, and how much we shall lay up for old age, and what one's charges per hour should be to make provision for these items, but where do our patients come in? It is just as resonable for them to tell us that they have every item of expense figured out and that only so much is allowed for dental services.

In reading Brother Bill's August letter, there is much which can be indorsed, and as a whole it is profitable and interesting reading, but some things smack of downright charlatanism; for instance, in his discussion about raising fees among other things regarding this case he says: "Mrs. Jones, I want to give this case a little different treatment, if you will let me. It will cost a little more, but it will be much better and even cheaper in the end." All this for the sole purpose of getting a larger fee. It reminds me of the "dental parlor," where amalgam fillings are twenty-five cents, silver fifty cents, and platinum one dollar, all from the same bottle. Suppose Mrs. Jones should say, "Why, doctor, I have always had implicit confidence in your integrity and ability, but if you have not heretofore given me your best services I have no reason to expect you will do so at an increased fee."

We all wish to live in fine houses, ride in automobiles, work a very limited number of hours a day and weeks a year, but the question is, will raising prices accomplish this. It has been said that when the Standard Oil Co. is fined, all they have to do is to raise the price of oil and make the people pay the price. According to the reasoning of Brother Bill, should one's living expenses increase, or should we need an extra amount for any purpose, all we have to do is to raise the price and make our patients pay it. This is possible if we have a monopoly, but quite a different proposition when it is only a few paces across the street to the dentist who is more moderate in his expenditures or who has the "Dental Parlor Bottle" to show his patients that which comes out of it according to their ability to pay. Prices, as a rule, will automatically arrange themselves without any explanation on our part as to why we charge more than formerly. All wish the best services they can get, and generally patronize those who can give them the best for the amount they are able to pay. If our clientele is among the rich, where price is no consideration, it is unnecessary for us to force it to be a consideration by making any excuse for charging a good fee, and if we work for the poor we cannot expect prices they are unable to pay. Good, honest work will slowly find its reward in a better clientele.

It is one's first duty to give our best services at all times, regardless of fees. If we do this and wish to work eight hours a day at, say, three dollars an hour, and there is a demand for the full eight hours, our prices may be easily increased; a few will drop out, but the character of our general clientele will be raised, and, as we had, at three dollars an hour, reached our limit, it gives us room to expand. But if at three dollars an hour there is a demand for our services of only four hours a day, we must look for other methods of increasing our income besides raising fees. No doubt all have noticed the inclination to say five dollars, instead of four, when our office is full—this is the principle of raising fees.—*Dental Digest.*

